# Workplace Psychosocial Resources and Risk of Sleep Disturbances Among Employees

**DOI:** 10.1001/jamanetworkopen.2023.12514

**Published:** 2023-05-09

**Authors:** Tianwei Xu, Reiner Rugulies, Jussi Vahtera, Sari Stenholm, Jaana Pentti, Linda L. Magnusson Hanson, Göran Kecklund, Jimmi Mathisen, Mads Nordentoft, Mika Kivimäki, Naja Hulvej Rod

**Affiliations:** 1 Stress Research Institute, Stockholm University, Stockholm, Sweden; 2 Department of Public Health, University of Copenhagen, Copenhagen, Denmark; 3 National Research Centre of the Working Environment, Copenhagen, Denmark; 4 Department of Psychology, University of Copenhagen, Copenhagen, Denmark; 5 Department of Public Health, University of Turku, Turku, Finland; 6 The Centre for Population Health Research, University of Turku, Turku, Finland; 7 Turku University Hospital, Turku, Finland; 8 Clinicum, Faculty of Medicine, University of Helsinki, Helsinki, Finland; 9 Finnish Institute of Occupational Health, Helsinki, Finland; 10 UCL Brain Sciences, University College London, London, United Kingdom; 11 Formerly with National Research Centre of the Working Environment, Copenhagen, Denmark; 12 Novo Nordisk, Copenhagen, Denmark

## Abstract

**Importance:**

Workplace psychosocial resources naturally tend to cluster in some work teams. To inform work-related sleep health promotion interventions, it is important to determine the associations between clustering of workplace resources and sleep disturbances when some resources are high while others are low and to mimic an actual intervention using observational data.

**Objective:**

To examine whether clustering of and changes in workplace psychosocial resources are associated with sleep disturbances among workers.

**Design, Setting, and Participants:**

This population-based cohort study used data from the Swedish Longitudinal Occupational Survey of Health (2012-2018), the Work Environment and Health in Denmark study (2012-2018), and the Finnish Public Sector Study (2008-2014), collected biennially. Statistical analysis was conducted from November 2020 to June 2022.

**Exposure:**

Questionnaires were distributed measuring leadership quality and procedural justice (ie, vertical resources) as well as collaboration culture and coworker support (ie, horizontal resources). Resources were divided into clusters of general low, intermediate vertical and low horizontal, low vertical and high horizontal, intermediate vertical and high horizontal, and general high.

**Main Outcomes and Measures:**

Odds ratios (ORs) and 95% CIs were reported from logistic regression models for the associations between the clustering of resources and concurrent and long-term sleep disturbances. Sleep disturbances were measured by self-administered questionnaires.

**Results:**

The study identified 114 971 participants with 219 982 participant-observations (151 021 [69%] women; mean [SD] age, 48 [10] years). Compared with participants with general low resources, other groups showed a lower prevalence of sleep disturbances, with the lowest observed in the general high group concurrently (OR, 0.38; 95% CI, 0.37-0.40) and longitudinally after 6 years (OR, 0.52; 95% CI, 0.48-0.57). Approximately half of the participants (27 167 participants [53%]) experienced changes in resource clusters within 2 years. Improvements in vertical or horizontal dimensions were associated with reduced odds of persistent sleep disturbances, and the lowest odds of sleep disturbances was found in the group with improvements in both vertical and horizontal dimensions (OR, 0.53; 95% CI, 0.46-0.62). A corresponding dose-response association with sleep disturbances was observed for decline in resources (eg, decline in both dimensions: OR, 1.74; 95% CI, 1.54-1.97).

**Conclusions and Relevance:**

In this cohort study of workplace psychosocial resources and sleep disturbances, clustering of favorable resources was associated with a lower risk of sleep disturbances.

## Key Points

**Question:**

What are the associations of clustering of and changes in workplace psychosocial resources (ie, leadership quality, procedural justice, culture of collaboration, and coworker social support) with sleep disturbances?

**Findings:**

In this cohort study, including 219 982 participant-observations nested within 114 971 participants, clustering of favorable workplace psychosocial resources was associated with a statistically significant lower risk of sleep disturbances. Improvements in leadership quality and procedural justice (ie, vertical resources) and in culture of collaboration and coworker social support (ie, horizontal resources) were associated with a lower risk of persistent sleep disturbances in a dose-response fashion.

**Meaning:**

These findings suggest that multilevel workplace interventions are essential to promote short- and long-term sleep quality among workers.

## Teaser

This cohort study examines whether clustering of and changes in workplace psychosocial resources are associated with sleep disturbances among workers concurrently and over the long term.

## Introduction

Sleep is essential to physiological restitution and recovery.^1,2^ Impaired sleep and prolonged activations of the physiological stress response may have far-reaching effects on the hormonal and immune system and metabolism, eventually contributing to a higher risk of cardiometabolic diseases.^3^ As the workplace is an important foundation for social and professional networks, investigating the preventive potential of favorable psychosocial work environment on reducing sleep disturbances is essential.^4^

Large-scale studies on workplace psychosocial resources, such as leadership quality, organizational justice, culture of collaboration, and coworker support, have only recently started to emerge. While higher workplace psychosocial resources have been associated with reduced risks of stress-related cardiometabolic diseases,^5,6^ previous findings on sleep disturbances are mixed. Some studies have reported a direct association between higher workplace psychosocial resources and lower risk of sleep disturbances among workers,^1,2,7,8,9,10,11^ while others could not identify such an association.^12,13,14^ In these studies, each workplace resources was addressed individually, while it is more likely that workplace psychosocial resources interact and thereby affect sleep and health through multiple mechanisms.^4^ Thus, it is important to investigate how different combinations of workplace psychosocial resources may be associated with sleep disturbances.

A further drawback in current evidence involves reliance on smaller-scale, cross-sectional findings.^1,2,7,8,9,10,11,12,13,14^ In samples of up to 3000 individuals, relational justice, leader support, and general workplace social support were associated with reduced risk of sleep disturbance within the first year of follow-up,^7,8^ but these associations disappeared with longer follow-up.^12,13,14^ Other studies with larger sample sizes supported a prospective association of relational justice and empowering leadership with sleep disturbances, but these studies did not consider the transition from short- to long-term.^9,10,11^ Importantly, such longitudinal associations may result from long-term effects of workplace psychosocial resources, but we cannot exclude the possibility of individual differences in perceptions of workplace characteristics and reporting sleep disturbances as an explanation for our results. Future studies emulating a target trial and investigating the extent to which changes in clustering of workplace resources are associated with changes in the risk of sleep disturbances are warranted to reduce the likelihood of this limitation and to evaluate the benefits of interventions with an aim to improve workplace resources.^15^ We are not aware of such studies having been conducted.

To address these limitations, we assessed the clustering of and changes in 4 major workplace psychosocial resources (ie, leadership quality, procedural justice, culture of collaboration, and coworker social support) to examine their concurrent and longitudinal associations with sleep disturbances. We studied 114 971 men and women from Denmark, Finland, and Sweden, with a up to 6 years of follow-up.

## Methods

### Study Population

This cohort study used data from the Work Environment and Health in Denmark (WEHD) study, Finnish Public Sector Study (FPS), and Swedish Longitudinal Occupational Survey of Health (SLOSH). Ethical approvals were obtained from the Regional Ethical Review Board in Stockholm (for use of SLOSH data)^16^ and the Ethics Committee of the Hospital District of Helsinki and Uusimaa (for use of FPS data).^17^ Use of WEHD data was approved by and registered with the Danish Data Protection Agency. Participants were informed about the purpose of the SLOSH, WEHD, and FPS, and by returning the questionnaire, the participants consented to participate. The study followed the Strengthening the Reporting of Observational Studies in Epidemiology (STROBE) reporting guideline.

The WEHD is a nationwide population-based biennial occupational survey, with baseline in 2012. It consists of individuals from the general working population and supplementary surveys on selected workplaces in Denmark.^18^ Data for this study were obtained from the 2012, 2014, and 2018 waves (wave 2016 was dropped due to sleep measurement inconsistencies).

The FPS is based on a dynamic public sector cohort in Finland followed up with repeated questionnaire surveys at 2- or 4-year intervals since 2000.^17^ Data for this study were obtained from the 2008, 2010, 2012, and 2014 surveys.

The SLOSH is a nationally representative cohort initiated in 2006 in Sweden, with follow-ups biennially. The participants of the cohort were recruited from the respondents of Swedish Working Environment Survey in 2003 to 2011.^16^ Data for this study were obtained from 2012, 2014, 2016 and 2018 in SLOSH. Except the 4-year interval between 2014 and 2018 for WEHD, all available cohort waves had a 2-year follow-up interval.

**Figure 1.**
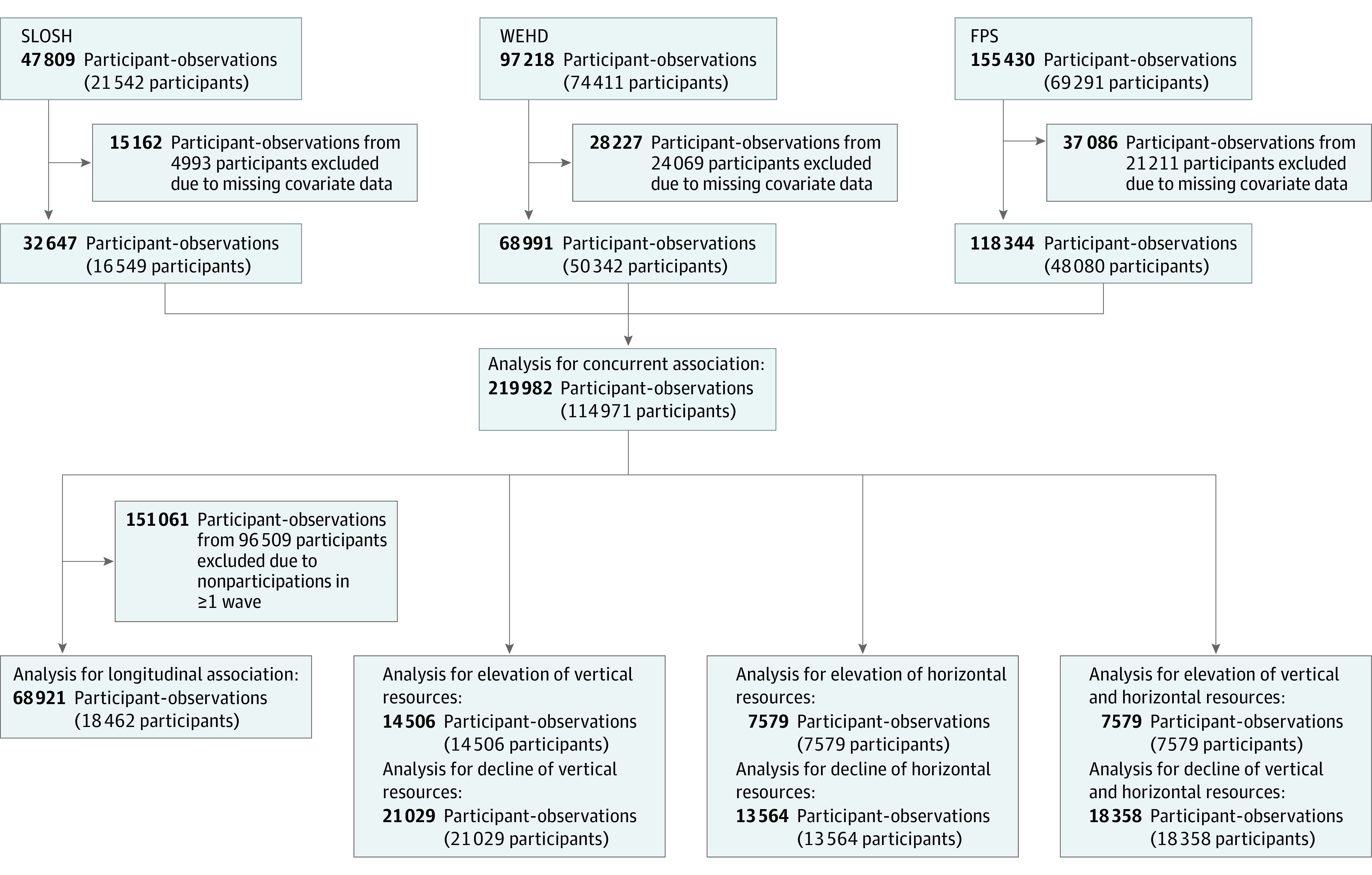
Flowchart of the Study and Analytical Populations FPS indicates Finnish Public Sector Study; SLOSH, Swedish Longitudinal Occupational Survey of Health; and WEHD, Work Environment and Health in Denmark.

For the concurrent analysis, we included participants who had information on workplace resources, sleep disturbances, and key confounders in 1 or more waves. For the longitudinal analysis, we further included only individuals who participated in all follow-up waves. For the analysis of changes in resources and changes in sleep, only participants of at least 2 consecutive waves were included. The flowchart of participants in each specific analysis is presented in Figure 1.

### Workplace Psychosocial Resources

We applied the framework of hierarchical dimensions of workplace psychosocial resources related to group-, leader-, and organizational-level variables,^4^ including culture of collaboration (horizontal), support from colleagues (horizontal), leadership quality (vertical), and procedural justice (vertical) (eTable 1 in Supplement 1). Horizontal resources are resources from the same hierarchical level.^19^ Vertical resources are resources from different hierarchical levels.^19^ All resources were measured using validated items and were categorized according to previous practice into 2 or 4 groups, while considering harmonization across cohorts (eAppendix 1 in Supplement 1).^6^

Culture of collaboration was dichotomized (low vs high) and was defined as the workplace collaborative efforts to achieve the best available results or to develop or apply new ideas. Coworker support was assessed as perceived colleagues’ support and was dichotomized (low vs high). The leadership variable included multiple dimensions, such as listening and appreciative, and was grouped into 4 levels (high, intermediately high, intermediately low, and low). The variable for procedural justice (ie, fairness in the principles and processes leading to decision-making) was also categorized into 4 groups (high, intermediately high, intermediately low, and low). Detailed descriptions of the variables can be found in eTable 1 in Supplement 1.^6^

Clustering of workplace psychosocial resources has been detected previously from these same cohorts using a latent class model.^6^ We used the same predefined 5-class categorization in this study: general low, intermediate vertical and low horizontal, low vertical and high horizontal, intermediate vertical and high horizontal, and general high (Table 1), because it showed better robustness across data waves, cohorts, and employment sectors compared with other categorizations (eAppendix 2 in Supplement 1).^6^ Changes of resources were defined as moving from 1 class to another across 2 consecutive waves, implying improvement or decline of either or both vertical and horizontal dimensions (eTable 2 in Supplement 1).

**Table 1.** Categorization of Workplace Psychosocial Resources Based on Previous Latent Class Analysis

Category	Vertical resources^a^	Horizontal resources^b^
General low	Low	Low
Intermediate vertical and low horizontal	Intermediately high or intermediately low	Low
Low vertical and high horizontal	Low	High
Intermediate vertical and high horizontal	Intermediately high or intermediately low	High
General high	High	High

^a^ Vertical resources include procedural justice and leadership quality.

^b^ Horizontal resources include colleagues’ support and culture of collaboration.

### Assessment of Sleep Disturbances

Sleep disturbances were defined as self-reported difficulties in initiating or maintaining sleep, nonrestorative sleep, or daytime tiredness during the past 4 weeks (for WEHD and FPS) or 3 months (for SLOSH) according to the Karolinska Sleep Questionnaire (3 or 4 items; for WEHD and SLOSH) or Jenkins Sleep Problem Scale (4 items; for FPS) (eTable 3 in Supplement 1). Sleep disturbances were identified if respondents reported any of the symptoms at least often or all the time (in WEHD), 2 to 4 nights per week (in FPS), or 3 to 4 nights per week (in SLOSH), and the same criteria were used for indicating insomnia.^10,20^ An alternative definition was applied to indicate the overall severity of the sleep disturbances (identified when the mean value was greater than or equal to the corresponding value of the aforementioned threshold).

### Covariates

Potential confounders were identified using directed acyclic graphs based on previous evidence,^21^ and included age, sex, country of birth (categorized as Scandinavian countries, other European countries, and other continents), marital status (married or cohabiting, single, separated or divorced, and widowed), highest educational level achieved (≤9, 10-12, and ≥13 years), type of employment contract (permanent, and nonpermanent), preexisting comorbidities, and preexisting mental disorders. Information on these variables were extracted from national registers in Denmark, Finland, and Sweden, except that marital status and employment contract in SLOSH and marital status in FPS were self-reported. Information on country of birth was not available in FPS. Preexisting comorbidities according to the Charlson Comorbidity Index and preexisting mental disorders were detected using the *International Classification of Diseases, Eighth Revision *(*ICD-8*), *International Classification of Diseases, Ninth Revision *(*ICD-9*), and *International Statistical Classification of Diseases and Related Health Problems, Tenth Revision *(*ICD-10*) codes from the national patient registries (eAppendix 3 in Supplement 1). Self-reported night shift work (yes and no) was also a potential confounder; however, night shift data were not available in FPS wave 2010 (wave 2).

### Statistical Analysis

Two different analytical approaches were used to determine the concurrent and longitudinal associations of clusters of workplace psychosocial resources with sleep disturbances and associations of changes in workplace psychosocial resources with changes in sleep disturbances (eFigure 1 in Supplement 1). In terms of missing values, we used complete-case analysis.

In the analysis for assessing the concurrent association between resources and sleep disturbances (eFigure 1 in Supplement 1), all available observations were included. To bridge the knowledge gap of varying follow-up intervals, we performed longitudinal analysis between workplace resources and sleep measured 2 and 6 years after the exposure measurement among individuals who participated in all follow-up waves (eFigure 1 in Supplement 1).

Next, we applied an emulated trial design to investigate the concurrent association between changes of resources and the risk of maintaining or developing sleep disturbances (eFigure 1 in Supplement 1).^15^ To address the issue of reverse causation, we applied a stricter restriction criteria to changes of resources occurring prior to changes of sleep disturbances. eTable 2 in Supplement 1 shows restrictions in each analysis.

We used multiple logistic regression models for the analyses. Some individuals were included in more than 1 baseline for the analysis of the concurrent association and the longitudinal analysis with the 2-year follow-up interval. Generalized estimating equations were used to account for the intraindividual correlation. All potential confounders were adjusted in all analyses, except that night work was adjusted for in a sensitivity analysis restricted to individuals with this information. All analyses were repeated for the alternative definition of sleep disturbances.

We used SAS statistical software version 9.4 (Proc Genmod; SAS Institute) to perform cohort-specific analyses. Given the small numbers of cohorts,^22^ fixed-effect meta-analyses were then performed to combine the cohort-specific estimates using R package meta version 4.16-2 (R Project for Statistical Computing). We used *I*^2^ statistics to quantify the cross-cohort heterogeneity. *P* values were 2-sided, and statistical significance was set at *P* = .05. Data were analyzed from November 2020 to June 2022.

## Results

A total of 114 971 participants with 219 982 participant-observations were included (mean [SD] age, 48 [10] years; 151 021 [69%] women). The 2 most common classes of work resources were intermediate vertical and high horizontal (55 608 participants [25%]) and general high (62 672 participants [28%]) were the 2 most common classes (Table 2).

**Table 2.** Demographic, Clinical, and Work-Related Characteristics of Participant-Observations Based on the Largest Available Sample of Participant-Observations

Workplace psychosocial resources	Participants, No. (%)					
	Total (n = 219 982)	General low (n = 30 515)	Intermediate vertical + low horizontal (n = 23 031)	Low vertical + high horizontal (n = 48 156)	Intermediate vertical + high horizontal (n = 55 608)	General high (n = 62 672)
**Demographic**						
Age, mean (SD), y	48 (10)	49 (9)	49 (9)	48 (10)	48 (10)	49 (10)
Gender						
Men	68 961 (31)	9051 (30)	6291 (27)	15 158 (31)	20 876 (38)	17 585 (28)
Women	151 021 (69)	21 464 (70)	16 740 (73)	32 998 (69)	34 732 (62)	45 087 (72)
Country of birth^a^						
Scandinavian	97 329 (96)	7477 (95)	5719 (93)	21 886 (97)	39 616 (96)	22 631 (95)
Other European countries	2464 (2)	229 (3)	213 (3)	462 (2)	928 (2)	632 (3)
Other continents	1845 (2)	171 (2)	207 (3)	308 (1)	694 (2)	465 (2)
Education level, y						
≤9	17 326 (8)	2566 (8)	1935 (8)	3534 (7)	4396 (8)	4896 (8)
>9-12	81 053 (37)	12 118 (40)	9030 (39)	17 935 (37)	20 370 (37)	21 599 (34)
≥13	121 603 (55)	15 831 (52)	12 066 (52)	26 687 (55)	30 842 (55)	36 177 (58)
Marital status						
Married	159 459 (72)	21 449 (70)	16 388 (71)	35 018 (73)	39 894 (72)	46 710 (75)
Single	33 335 (15)	4743 (16)	3379 (15)	7348 (15)	9709 (17)	8156 (13)
Separated	23 831 (11)	3871 (13)	2867 (12)	5084 (11)	5268 (9)	6741 (11)
Widowed	3357 (2)	452 (1)	397 (2)	706 (1)	737 (1)	1065 (2)
**Clinical**						
Comorbidity score, mean (SD)	0.20 (0.64)	0.19 (0.58)	0.19 (0.59)	0.22 (0.64)	0.21 (0.71)	0.19 (0.61)
Mental disorders	4746 (2)	784 (3)	530 (2)	1033 (2)	1186 (2)	1213 (2)
**Work-related **						
Temporary contract	14 266 (6)	1749 (6)	1740 (8)	3162 (7)	3388 (6)	4227 (7)
Night shifts^a^	20 078 (9)	3853 (13)	2245 (10)	4786 (10)	4746 (9)	4448 (7)

^a^ Data were missing for 118 344 participant-observations for country of birth and 28 873 participant-observations for shiftwork.

### Concurrent and Longitudinal Associations Between Resources and Sleep

**Figure 2.**
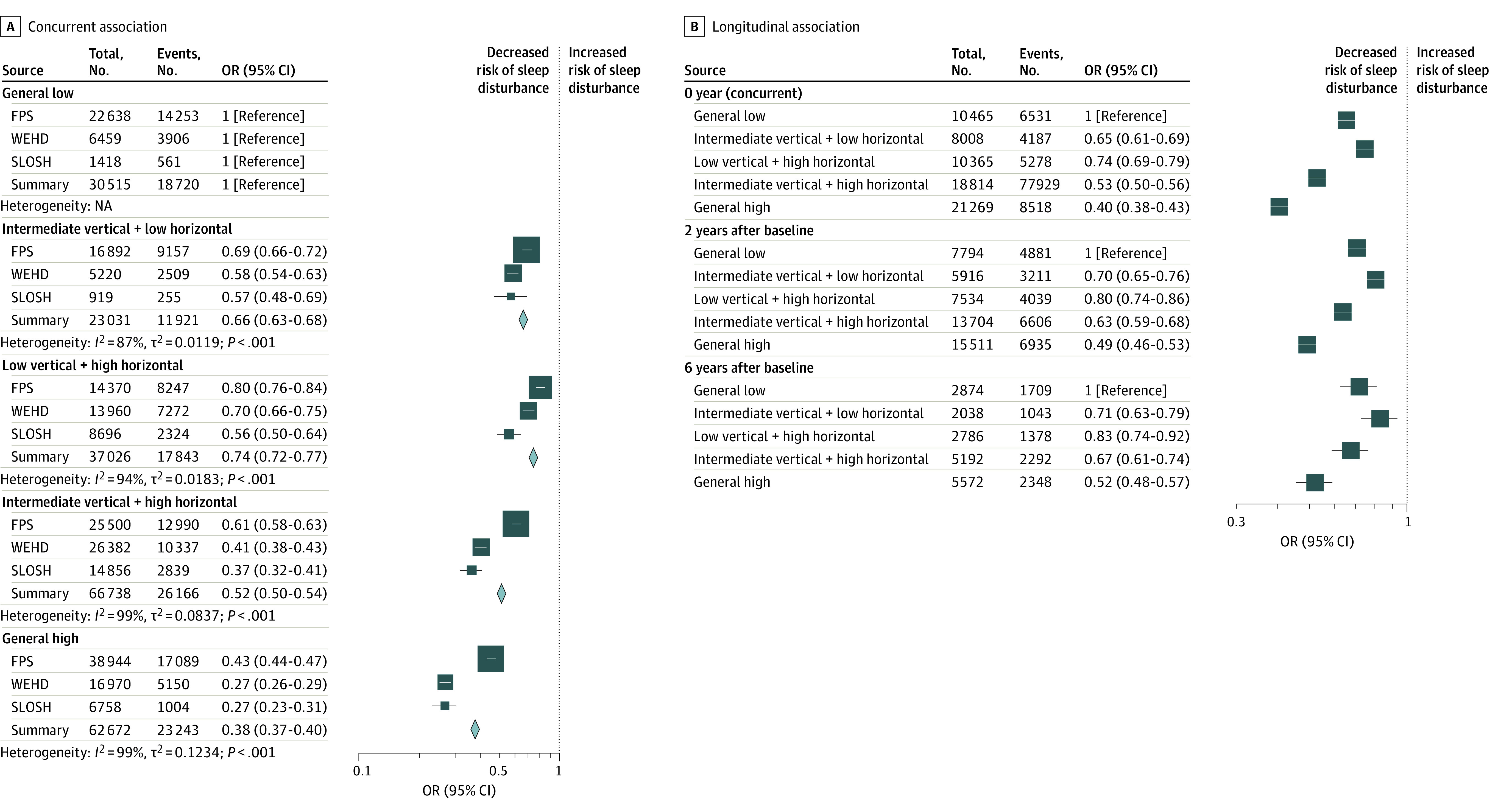
Concurrent and Longitudinal Associations Between Workplace Psychosocial Resources and Sleep Disturbances FPS indicates Finnish Public Sector Study; NA, not applicable; OR, odds ratio; SLOSH, Swedish Longitudinal Occupational Survey of Health; and WEHD, Work Environment and Health in Denmark.

Using the largest available sample size (114 971 participants), we found relatively stable trajectories in the prevalence of disturbed sleep according to resource classes and cohorts (eFigure 2 in Supplement 1). In the confounder-adjusted model, compared with the general low resource class, having a general high level of resources was associated with an overall lower prevalence of sleep disturbances (odds ratio [OR], 0.38; 95% CI, 0.37-0.40). Although there was substantial cross-cohort heterogeneity (*I*^2^ > 80%), all cohort-specific estimates pointed to the same direction (Figure 2A).

A total of 18 462 individuals participated in all waves. Among 7491 individuals with baseline sleep disturbances, 5414 individuals (72%) reported sleep disturbances 2 years later, and 3095 individuals (41%) reported sleep disturbances after 6 years. When comparing general high with general low resources, the association between clustering of resources and sleep disturbances remained pronounced after 2 years (OR, 0.49; 95% CI, 0.46-0.53) and 6 years (OR, 0.52; 95% CI, 0.48-0.57) (Figure 2B).

There was a dose-response pattern for vertical resources when horizontal resources were high (Figure 2B). However, observing the contrast between intermediate vertical and low horizontal vs intermediate vertical and high horizontal, the role of horizontal resources was less clear in the presence of an intermediate level of vertical resources in the concurrent analysis (OR, 0.82; 95% CI, 0.75-0.89), and it lost statistical significance after 2 years (OR, 0.90; 95% CI, 0.81-1.00) and 6 years (OR, 0.94; 95% CI, 0.81-1.10). Nevertheless, when vertical resources were low, perceiving high horizontal resource was associated with a lower risk of sleep disturbances from the concurrent analysis to 6 years of follow-up (Figure 2B).

### Association Between Changes in Resources and Changes in Sleep Disturbances

**Figure 3.**
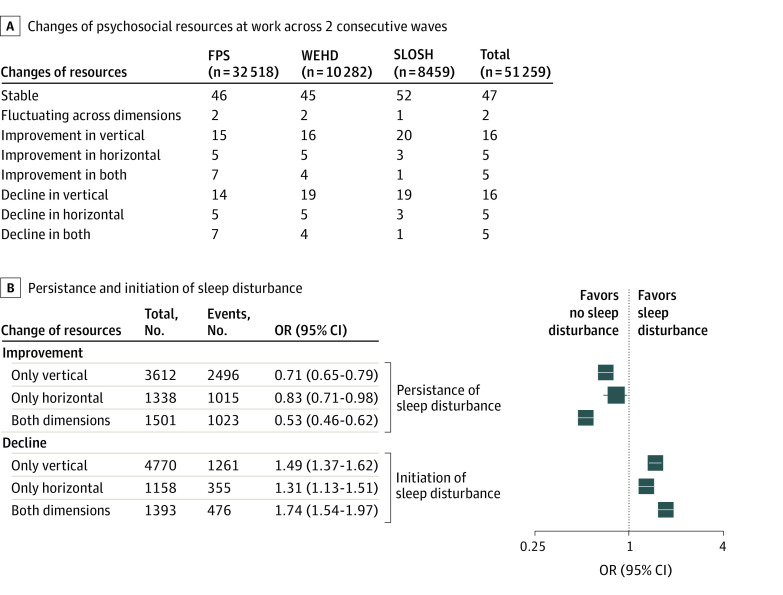
Association Between Changes in Resources and Changes of Sleep Disturbances FPS indicates Finnish Public Sector Study; OR, odds ratio; SLOSH, Swedish Longitudinal Occupational Survey of Health; and WEHD, Work Environment and Health in Denmark.

Among 51 259 employees participating 2 consecutive waves, 27 167 (53%) experienced a change in resources across 2 consecutive follow-ups (Figure 3A). Changes in vertical resources accounted for most of the change, including 8233 employees (16%) who experienced improvement and 8130 employees (16%) who experienced decline.

In the analyses of improvement (Figure 3B), 72% to 75% of employees reported persisting sleep problems between baselines and follow-up. Compared with the stable resource group, the participants who experienced improvement in either vertical or horizontal had reduced odds of sleep disturbance (vertical improvement: OR, 0.71; 95% CI, 0.65-0.79; horizontal improvement: OR, 0.83; 95% CI, 0.71-0.98; improvement on both dimensions: OR, 0.53; 95% CI, 0.46-0.62).

In the analyses of declined resources (Figure 3B), nearly one-fourth of employees developed sleep disturbances from baseline to follow-up. Individuals who experienced a decline in both vertical and horizontal resource dimensions had the highest odds of developing sleep disturbances (OR, 1.74; 95% CI, 1.54-1.97) compared with individuals with unchanged resources. A decline in vertical or horizontal resources were also associated with higher odds of developing sleep disturbances (vertical decline: OR, 1.49; 95% CI, 1.37-1.62; horizontal decline: 1.31; 95% CI, 1.13-1.51), which suggests there may have been an additive pattern across different resource dimensions.

Similar findings were observed when changes in resources occurred before changes in sleep disturbances. However, these temporal associations were weaker, less precisely estimated, and did not achieve statistical significance at conventional levels (eFigure 3 in Supplement 1).

### Sensitivity Analyses

Additional adjustment for night shifts did not materially change the results (eFigure 3 and eFigure 4 in Supplement 1). Results were similar when applying the alternative definition of sleep disturbances (eFigure 5 in Supplement 1).

## Discussions

The findings of this cohort study, including data on 219 982 participant-observations from 114 971 participants in 3 large longitudinal cohorts, suggest that better psychosocial resources at work were associated with lower risk of sleep disturbances, both concurrently and longitudinally, with vertical resources of greater importance in the long term. Supporting modifiability of sleep disturbance risk by targeting workplace psychosocial resources, we observed that improvements in workplace psychosocial resources were associated with a reduced risk of persisting sleep disturbances and deterioration of resources was associated with the development of sleep disturbances.

Our findings are generally in line with several previous smaller-scale studies,^1,2,7,9,10,11^ and add to this literature by examining clustering of vertical and horizontal psychosocial resources at work with an emulated trial design in observational data. We demonstrate that the clustering of vertical and horizontal resources is important to consider for long-term sleep patterns and that ignoring clustering of different hierarchical level of resources may partly explain the lack of association between resources and sleep, as some of the associations seem to be synergistic.^12,13,14^ Moreover, our findings on changes in resources confirm findings from a single-cohort study, suggesting an association between changes in relational justice and changes in sleep.^10^

According to the effort-recovery model, improved psychosocial resources from work could contribute to a better switch-off from work, in turn leading to better recovery process from work, including improved sleep.^23^ Conversely, a decline in workplace resources can be a stressor that can potentially trigger dysregulation of the hypothalamic-pituitary-adrenal and sympathetic-adreno-medullar axes, which can adversely affect sleep.^2^ Other potential mechanistic pathways involve reductions in potential consequences of low psychosocial resources, such as workplace bullying, mental health problems, and problematic health-related behaviors.^24,25,26^

Our findings suggest that vertical resources might be more crucial than horizontal resources in associations with long-term sleep disturbances. In the presence of high horizontal resources, vertical resources showed a long-term dose-response association, while the same association was not observed for horizontal resources, given an intermediate level of vertical resources was present. This finding is plausible, considering the greater power of leaders to affect a positive working environment,^4^ such that vertical resources may promote horizontal resources in the long term.^27^ A study by Framke et al^28^ suggested workplace horizontal social capital may be particularly relevant for sleep disturbances; however, their study was based on a smaller occupation-specific sample. Future investigations of the interactive cross-dimensional mechanisms of work resources may facilitate the design of cost-effective strategies for the prevention of sleep disturbances.

The concept of workplace psychosocial resources overlaps a number of occupational and organizational theories, including the demand-control model, the job-demand resource model, the effort-reward imbalance model, and the concept of organizational justice.^29,30^ A 2017 study by Nielsen et al^4^ proposed that workplace resources at the individual (eg, psychological capital), group (eg, team climate), leader (eg, leadership quality), and organizational levels (eg, perceived organizational support and justice) may be associated with better job performance and well-being among employees. Following this framework, we have shown that vertical (organization or leader) and horizontal (the work group) clusters of resources were associated with health-related outcomes.^5,6^ Our findings further highlight the potential benefits of multilevel workplace interventions in promoting sleep of employees.

### Limitations and Strengths

This study has some limitations. We applied a novel clustering of workplace resources based on predefined information on 4 important workplace resources. Thus, caution should be taken when generalizing our findings to other workplace resources, such as job control, rewards, and autonomy.

We included more than 100 000 participants with 6 years of follow-up measured up to 3 times and reported results from both concurrent and longitudinal analyses. Although we applied an emulated trial design, reverse causation cannot be fully ruled out due to lack of statistical power to draw solid conclusions on temporality. Furthermore, it is likely that sleep, stress, and workplace resources are affecting each other in a dynamic manner, which may create feedback loops over time. Future studies with larger sample sizes are needed to disentangle these intertwined mechanisms. Our population-based sample included employees from different occupational groups, industries, and employment types. Although point estimates varied substantially across these cohorts, most cohort-specific results pointed to the same direction, implying the validity and generalizability across countries and different settings. The data sets were only followed until 2018, which is before the COVID-19 pandemic began. Therefore, future studies are needed to understand whether, for example, the COVID-19–related changes in work-from-home opportunities would modify the observed associations. Other potential modifiers, including age group, gender, employment sector, educational level, and occupational grade, should be explored in future studies to detect at-risk groups that would benefit most from interventions.

With a large sample size and repeated follow-ups, we assessed the general association between resources and sleep as an extension to prior studies, allowing for direct comparisons with previous findings addressing different follow-up lengths and observing short- to long-term transitions. Five combinations of vertical and horizontal dimensions of resources enabled us to weigh the importance of 1 dimension at a time, with or without the presence of the other. A further step was taken to analyze the associations with changes in resources, mimicking a trial, which can be informative for intervention development when data from an actual trial are not available.

## Conclusions

In this cohort study including 3 Scandinavian cohorts, nearly half of the employees experienced a low level of 1 or more vertical or horizontal dimensions of workplace psychosocial resources, which included procedural justice, leadership quality, coworker support, and culture of collaboration. Favorable resources at work were associated with a lower risk of sleep disturbances, and improvements in resources were associated with lower risk of sleep disturbances. Our findings justify future intervention studies to examine the extent to which improvements in workplace psychosocial resources could facilitate remission or recovery from sleep disturbances and prevent development, deterioration, or prolongation of sleep disturbances among employees.

## Notes

Supplement 1. **eAppendix 1.** The Choice of Measurements, Dimensions, and Cutoffs of Workplace Psychosocial Resources **eAppendix 2.** Comparisons with Other Possible Categorizations **eAppendix 3.** Measurement of Covariates **eTable 1.** Items Measuring Workplace Resources **eTable 2.** Changes of Resources and Restrictions **eTable 3.** Measurement Invariances for Sleep Measurement Across Waves, Using the Confirmatory Factor Analysis **eFigure 1.** Study Design **eFigure 2.** Trajectory of Sleep Disturbances **eFigure 3.** Results From Analyses of Changes, Before and After the Adjustment of Night Shifts **eFigure 4.** Additional Adjustment for Night Shifts for Analyses on Concurrent and Longitudinal Associations **eFigure 5.** Results From Alternative Definition of Sleep Disturbances

Supplement 2.
Data Sharing Statement
