# Error in Author Affiliation

**DOI:** 10.1001/jamanetworkopen.2023.22243

**Published:** 2023-06-21

In the Original Investigation titled “Workplace Psychosocial Resources and Risk of Sleep Disturbances Among Employees,”^1^ published May 9, 2023, there was an error in the affiliations provided for Mads Nordentoft, PhD. Dr Nordentoft should have been listed as affiliated with the National Research Centre for the Working Environment, Copenhagen, Denmark. This article has been corrected.^1^
