# Supplementary material for: Workplace Psychosocial Resources and Risk of Sleep Disturbances Among Employees
Source: JAMA Netw Open. 2023 May 9;6(5):e2312514. doi: 10.1001/jamanetworkopen.2023.12514 (PMC10170336; doi:10.1001/jamanetworkopen.2023.12514)
Supplement: Supplement 1. — eAppendix 1. The Choice of Measurements, Dimensions, and Cutoffs of Workplace Psychosocial Resources eAppendix 2. Comparisons with Other Possible Categorizations eAppendix 3. Measurement of Covariates eTable 1. Items Measuring Workplace Resources eTable 2. Changes of Resources and Restrictions eTable 3. Measurement Invariances for Sleep Measurement Across Waves, Using the Confirmatory Factor Analysis eFigure 1. Study Design eFigure 2. Trajectory of Sleep Disturbances eFigure 3. Results From Analyses of Changes, Before and After the Adjustment of Night Shifts eFigure 4. Additional Adjustment for Night Shifts for Analyses on Concurrent and Longitudinal Associations eFigure 5. Results From Alternative Definition of Sleep Disturbances [file jamanetwopen-e2312514-s001.pdf]

Supplemental Online Content

Xu T, Rugulies R, Vahtera J, et al. Workplace psychosocial resources and risk of sleep disturbances among employees. *JAMA Netw Open*. 2023;6(5):e2312514. doi:10.1001/jamanetworkopen.2023.12514

eAppendix 1. The Choice of Measurements, Dimensions, and Cutoffs of Workplace Psychosocial Resources

eAppendix 2. Comparisons with Other Possible Categorizations

eAppendix 3. Measurement of Covariates

eTable 1. Items Measuring Workplace Resources

eTable 2. Changes of Resources and Restrictions

eTable 3. Measurement Invariances for Sleep Measurement Across Waves, Using the Confirmatory Factor Analysis

eFigure 1. Study Design

eFigure 2. Trajectory of Sleep Disturbances

eFigure 3. Results From Analyses of Changes, Before and After the Adjustment of Night Shifts

eFigure 4. Additional Adjustment for Night Shifts for Analyses on Concurrent and Longitudinal Associations

eFigure 5. Results From Alternative Definition of Sleep Disturbances

This supplemental material has been provided by the authors to give readers additional information about their work.

© 2023 Xu T et al. *JAMA Network Open.*

eAppendix 1. The Choice of Measurements, Dimensions, and Cutoffs of Workplace Psychosocial Resources

The measurement on support from colleagues was derived from the Statistics Finland scale on the working climate.[1] Similar item (e.g. "my colleagues are there for me") has been used in another multicohort study on type 2 diabetes, including cohorts from Sweden, United Kingdom and France.[2] We applied the exact same cut-off. Measurement on collaboration is part of the measurement of workplace social capital scale,[3] which has been used to investigate the association with hypertension and mortality.[4,5] We used the median separation applied in papers for workplace social capital. The measurement of procedural justice (Moorman scale) has been applied previously in different contexts, e.g. in Sweden (quartile separation)[6] and in Finland (tertile separation).[7] Because this study is part of a larger research programme containing other cohorts, for cohort harmonization, we selected four dimensions for leadership due to data availability (three questions from The Stress Profile[8] and one individual item on managers listening from relational justice 9), following previous practice in another multicohort study.10 In the previous practice leadership was dichotomised into "good" and "poor" leadership quality according to the median value of the constructed scale. We further decided to use the quartile separation for both procedural justice and leadership quality, to allow a more consistent interpretation of the meaning and at the same time follow the previous practice.

References:

1. Lehto A-M. Quality of working life and equity. Helsinki, Finland: Statistics Finland. 1991.
2. Mortensen J, Clark AJ, Lange T, et al. Informal caregiving as a risk factor for type 2 diabetes in individuals with favourable and unfavourable psychosocial work environments: A longitudinal multi-cohort study. Diabetes & Metabolism. 2018; 44 (1): 38-44.
3. Kouvonen A, Kivimäki M, Vahtera J, et al. Psychometric evaluation of a short measure of social capital at work. BMC public health. 2006; 6 (1): 251.
4. Oksanen T, Kawachi I, Jokela M, et al. Workplace social capital and risk of chronic and severe hypertension: a cohort study. Journal of hypertension. 2012; 30 (6): 1129-1136.
5. Oksanen T, Kivimäki M, Kawachi I, et al. Workplace social capital and all-cause mortality: A prospective cohort study of 28 043 public-sector employees in Finland. American journal of public health. 2011; 101 (9): 1742-1748.
6. Persson V, Eib C, Bernhard-Oettel C, Leineweber C. Effects of procedural justice on prospective antidepressant medication prescription: a longitudinal study on Swedish workers. BMC public health. 2020; 20: 1-9.
7. Lallukka T, Halonen JI, Sivertsen B, et al. Change in organizational justice as a predictor of insomnia symptoms: longitudinal study analysing observational data as a non-randomized pseudo-trial. International journal of epidemiology. 2017; 46 (4): 1277-1284.
8. Setterlind S, Larsson G. The stress profile: A psychosocial approach to measuring stress. Stress Medicine. 1995; 11 (1): 85-92.
9. Elovainio M, Kivimäki M, Vahtera J. Organizational justice: evidence of a new psychosocial predictor of health. American journal of public health. 2002; 92 (1): 105-108.
10. Madsen IEH, Hanson LLM, Rugulies R, et al. Does good leadership buffer effects of high emotional demands at work on risk of antidepressant treatment? A prospective study from two Nordic countries. Social psychiatry and psychiatric epidemiology. 2014; 49 (8): 1209-1218.

© 2023 Xu T et al. *JAMA Network Open.*

eAppendix 2. Comparisons with Other Possible Categorizations

We selected a five-class model based on the Bayesian Information Criterion ([BIC], a lower value of model fit indicates a better fit with the data), distribution of class membership probabilities, class sizes and interpretability of the classes. In determination of the class model we also ensured the comparability of the model across cohorts.

**Cohort**	**BIC for 2 classes**	**BIC for 3 classes**	**BIC for 4 classes**	**BIC for 5 classes**	**BIC for 6 classes**
WEHD	337100.2	330751.1	328800.2	328165.6	Not convergent
FPS	486247.4	483256.1	480956.2	480704.7	Not convergent
SLOSH	108518.6	107530.1	107611.4	107607.6	107671.1
Abbreviation: BIC= Bayesian Information Criterion, the lower the better.					

BIC decreased consistently from the 2-class model to 5-class model and when adding a sixth class. Models in WEHD and FPS did not converge and BIC for 6 classes in SLOSH was larger than that for 5 classes. In light of these findings, the 5-class model provides the best model, which also shows very good comparability across the cohorts.

© 2023 Xu T et al. *JAMA Network Open.*

eAppendix 3. Measurement of Covariates

Information on key covariates were extracted from the national register in Denmark, Finland and Sweden, except that marital status in Finland and employment contract in Sweden were measured by self-report. Pre-existing comorbidities according to Charlson Comorbidity Index (table below) and mental disorders were detected using ICD codes from national patient register.

ICD10 for diagnosed mental disorders: F01-F99; ICD 8 for diagnosed mental disorders: 290-315.

ICD codes for Charlson Comorbidity Index

Condition	Weights	ICD-8	ICD-9	ICD-10
Acute myocardial infarction	1	410	410, 412	I21, I22, I252
Congestive heart failure	1	427.09; 427.10; 427.11; 427.19; 428.99; 782.49	428	I50
Peripheral vascular disease	1	440; 441; 442; 443; 444; 445	441, 4439, 7854, V434	I71, I790, I739, R02, Z958, Z959
Cerebral vascular accident	1	430–438	430–438	I60, I61, I62, I63, I65, I66, G450, G451, G452, G458, G459, G46, I64, G454, I670, I671, I672, I674, I675, I676, I677, I678, I679, I681, I682, I688, I69
Dementia	1	290.09–290.19; 293.09	290	F00, F01, F02, F051
Pulmonary disease	1	490–493; 515–518	490, 491, 492, 493, 494, 495, 496, 500, 501, 502, 503, 504, 505	J40, J41, J42, J44, J43, J45, J46, J47, J67, J44, J60, J61, J62, J63, J66, J64, J65
Connective tissue disorder	1	712; 716; 734; 446; 135.99	7100, 7101, 7104, 7140, 7141, 7142, 71481(now 5171), 725	M32, M34, M332, M053, M058, M059, M060, M063, M069, M050, M052, M051, M353
Peptic ulcer	1	530.91; 530.98; 531–534	531, 532, 533, 534	K25, K26, K27, K28
Liver disease	1	571; 573.01; 573.04	5712, 5714, 5715, 5716	K702, K703, K73, K717, K740, K742, K746, K743, K744, K745
Diabetes	1	249.00; 249.06; 249.07; 249.09 250.00; 250.06; 250.07; 250.09	2500,2501, 2502, 2503, 2507	E109, E119, E139, E149, E101, E111, E131, E141, E105, E115, E135, E145
Diabetes complications	2	249.01–249.05; 249.08 250.01–250.05; 250.08	2504, 2505, 2506	E102, E112, E132, E142 E103, E113, E133, E143 E104, E114, E134, E144
Paraplegia	2	344	342, 3441	G81 G041, G820, G821, G822
Renal disease	2	403; 404; 580–583; 584; 590.09; 593.19; 753.10–753.19; 792	582, 5830, 5831, 5832, 5833, 5835, 5836, 5837, 5834, 585, 586, 588	N03, N052, N053, N054, N055, N056, N072, N073, N074, N01, N18, N19, N25
Cancer	2	140–194<br>204–207<br>200–203; 275.59	14, 15, 16, 18, 170, 171, 172, 174, 175, 176, 179, 190, 191, 192, 193, 194, 1950, 1951, 1952, 1953, 1954, 1955, 1958, 200, 201, 202, 203, 204, 205, 206, 207, 208	C0, C1, C2, C3, C40, C41, C43, C45, C46, C47, C48, C49, C5, C6, C70, C71, C72, C73, C74, C75, C76, C80, C81, C82, C83, C84, C85, C883, C887, C889, C900, C901, C91, C92, C93, C940, C941, C942, C943, C9451, C947, C95, C96
Metastatic cancer	6	195–198; 199	196, 197, 198, 1990, 1991	C77, C78, C79, C80
Severe liver disease	3	070.00; 070.02; 070.04; 070.06; 070.08; 573.00; 456.00–456.09	5722, 5723, 5724, 5728	K729, K766, K767, K721
HIV	6	79.83	042, 043, 044	B20, B21, B22, B23, B24

© 2023 Xu T et al. *JAMA Network Open.*

eTable 1. Items Measuring Workplace Resources

Type of resource	WEHD	FPS	SLOSH
**Support from colleagues**	How often do you and your colleagues collaborate when you are facing problems that require a solution?<br><br>(5 point likert scale)<br><br>'All the time' or 'often',<br><br>Vs. 'sometimes', 'rarely' or 'never'	How much do you think your teammates support and help you?<br><br>(5 point likert scale)<br><br>'Very much', 'a lot',<br><br>Vs. 'neither much nor a little', 'a little', 'little'	My colleagues are there for me.<br><br>(4 point likert scale)<br><br>'Totally agree', or 'somewhat agree',<br><br>Vs. 'somewhat disagree', 'totally disagree'
**Culture of collaboration**	How often do you and your colleagues help each other for achieving the best possible results?<br><br>(5 point likert scale)<br><br>'All the time' or 'often',<br><br>Vs. 'sometimes', 'rarely' or 'never'	1. Do members of the work unit build on each other's ideas in order to achieve the best possible outcome?<br>2. People in the work unit cooperate in order to help develop and apply new ideas.<br>(Cronbach's alpha=0.77)<br>(5 point likert scale)<br><br>'Very much', 'a lot', 'neither much nor a little', 'a little', 'little'<br><br>Mean seperation	1. Members of the work unit build on each other's ideas in order to achieve the best possible outcome.<br>2. People in the work unit cooperate in order to help develop and apply new ideas.<br>(Cronbach's alpha=0.87-0.88)<br>(4 point likert scale)<br><br>'Totally agree', or 'somewhat agree', 'somewhat disagree', 'totally disagree'<br><br>Mean separation
**Leadership quality**	How often:<br><br>1. does your immediate manager explain the company's objectives, so you understand what they mean for your work tasks?<br>2. do you have sufficient authority in relation to your responsibilities at work?<br>3. does your immediate manager take the time to engage in your professional development?<br>4. does your immediate manager involve you in the planning of your work?<br>5. does your immediate manager give you the necessary feedback (praise and criticism) for your work?<br>6. is your work recognized and appreciated by the management?<br>7. do you get the necessary help and support from your immediate manager?<br>8. can you trust the information that comes from the management?<br><br>(Cronbach's alpha=0.89)<br><br>(5 point likert scale)<br><br>'All the time', 'often', 'sometimes', 'rarely', 'never'<br><br>Mean seperation	1. Our superior does not care about the feelings of the employees.<br>2. Our superior listens to his subordinates' opinions in important cases.<br>3. Our superior rewards good work effort.<br>4. Our superior informs us in good time on decisions taken and their consequences.<br><br>(Cronbach's alpha=0.88)<br><br>(5 point likert scale)<br><br>'Totally agree', 'somewhat agree', 'not agree nor disagree', 'somewhat disagree', 'totally disagree'<br><br>Mean seperation	1. My boss shows that he/she cares how things are for me and how I feel.<br>2. Your manager genuinely listen to what you have to say.<br>3. I get the acknowledgement I deserve from my superiors.<br>4. My boss gives me the information I need.<br><br>(Cronbach's alpha=0.84)<br><br>(4 point likert scale)<br><br>'Totally agree', 'somewhat agree', 'somewhat disagree', 'totally disagree'<br><br>Mean seperation
**Procedural Justice**	How often the concerns of all those affected by the decision were heard.<br><br>(5 point likert scale)<br><br>'All the time'<br><br>Vs. 'often', or 'sometimes',<br><br>Vs. 'rarely',<br><br>Vs. 'never'	1. Procedures are designed to collect accurate information necessary for making decisions.<br>2. Procedures are designed to provide opportunities to appeal or challenge the decision.<br>3. Procedures are designed to hear the concerns of all those affected by the decision.<br>4. Procedures are designed to generate standards so that decisions can be made with consistency.<br><br>(Cronbach's alpha=0.90)<br><br>(5 point likert scale)<br><br>'Totally agree', 'somewhat agree', 'not agree nor disagree', 'somewhat disagree', 'totally disagree'<br><br>Mean seperation	1. Decisions are taken on the basis of correct information.<br>2. Bad decisions can be revoked or changed.<br>3. All sides affected by the decision are represented.<br>4. Decisions taken are consistent (the same rules apply to everyone).<br>5. Everyone is entitled to give their opinion in matters of immediate personal concern.<br>6. Feedback is provided regarding the consequences of decisions and people are informed accordingly.<br>7. It is possible to obtain a more detailed account of the information that underlies decisions, if needed.<br><br>(Cronbach's alpha=0.91)<br><br>(5 point likert scale)<br><br>'Totally agree', 'somewhat agree', 'neither agree nor disagree', 'somewhat disagree', 'totally disagree'<br><br>Mean seperation

© 2023 Xu T et al. *JAMA Network Open.*

eTable 2. Changes of Resources and Restrictions

**Changes in resources**	**Definition of change between T_x and T_{x+1}**	**Restrictions at T_x for the change of concern (Concurrent and temporal changes)**	**Restrictions at T_{x+1} for the change of concern (Temporal changes)**
Stable	Same resources between T_x and T_{x+1}		
Fluctuating resources across dimensions	Intermediate vertical + low horizontal → Low vertical + high horizontal<br>Low vertical + high horizontal → Intermediate vertical + low horizontal	-	-
Improvement of vertical	General low → Intermediate vertical + low horizontal<br>Low vertical + high horizontal → Intermediate vertical + high horizontal<br>Low vertical + high horizontal → General high	Resource = ‘General low’ or ‘Low vertical & high horizontal’<br>Disturbed sleep	Disturbed sleep
Improvement of horizontal	General low → Low vertical + high horizontal<br>Intermediate vertical + low horizontal → Intermediate vertical + high horizontal	Resource = ‘General low’ or ‘Intermediate vertical & low horizontal’<br>Disturbed sleep	Disturbed sleep
Improvement of both vertical and horizontal	General low → Intermediate vertical + high horizontal<br>General low → General high<br>Intermediate vertical + low horizontal → General high	Resource = ‘General low’ or ‘Intermediate vertical & low horizontal’<br>Disturbed sleep	Disturbed sleep
Decline of vertical	Intermediate vertical + low horizontal → General low<br>Intermediate vertical + high horizontal → Low vertical + high horizontal<br>General high → Low vertical + high horizontal	Resource = ‘Intermediate vertical & low horizontal’ or ‘General high’<br>Normal sleep	Normal sleep
Decline of horizontal	Low vertical + high horizontal → General low<br>Intermediate vertical + high horizontal → Intermediate vertical + low horizontal	Resource = ‘Low vertical & high horizontal’ or ‘Intermediate vertical & high horizontal’<br>Normal sleep	Normal sleep
Decline of vertical and horizontal	Intermediate vertical + high horizontal → General low<br>General high → General low<br>General high → Intermediate vertical + low horizontal	Resource = ‘Intermediate vertical & high horizontal’ or ‘General high’<br>Normal sleep	Normal sleep

© 2023 Xu T et al. *JAMA Network Open.*

eTable 3. Measurement Invariances for Sleep Measurement Across Waves, Using the Confirmatory Factor Analysis

Cohort	Invariance testing levels	Chi-square (df)	Comparative Fit Index
SLOSH	Free all parameters	405.5 (8)	0.992
SLOSH	Metric invariance -- loadings invariant	442.5 (17)	0.992
SLOSH	Scalar invariance -- loadings & intercepts invariant	490.1 (26)	0.991
WEHD	Free all parameters	0 (Saturated model)	1.000
WEHD	Metric invariance -- loadings invariant	10.6 (4)	1.000
WEHD	Scalar invariance -- loadings & intercepts invariant	206.7 (8)	0.997
FPS	Free all parameters	2587.5 (8)	0.988
FPS	Metric invariance -- loadings invariant	2683.6 (17)	0.988
FPS	Scalar invariance -- loadings & intercepts invariant	2911.4 (26)	0.987

Due to the large sample size, we evaluated the existence of measurement invariance using fit indices, such as the comparative fit index (CFI). Using the criteria suggested by Meade and his colleagues (ΔCFI ≤0.002 for metric invariance and ΔCFI ≤0.01 for scalar invariances),[11] the results showed measurement invariance at all testing levels in every cohort.

Reference:

11. Meade AW, Johnson EC, Braddy PW. Power and sensitivity of alternative fit indices in tests of measurement invariance. Journal of applied psychology. 2008;93(3):568.

© 2023 Xu T et al. *JAMA Network Open.*

eFigure 1. Study Design

Conceptual models for concurrent and longitudinal associations with various follow-up lengths (A) and changes of workplace psychosocial resources on changes of sleep disturbances (B)

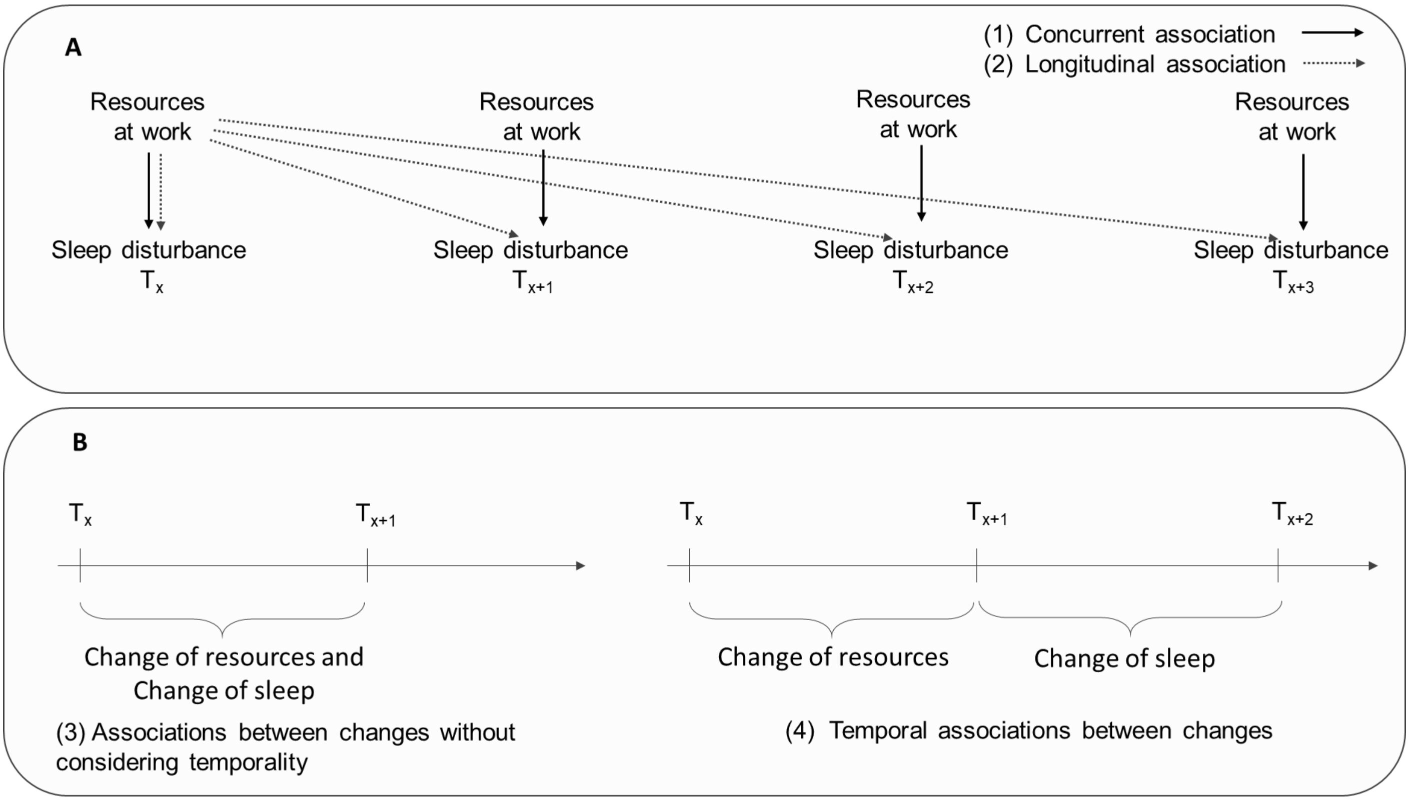

eFigure 2. Trajectory of Sleep Disturbances

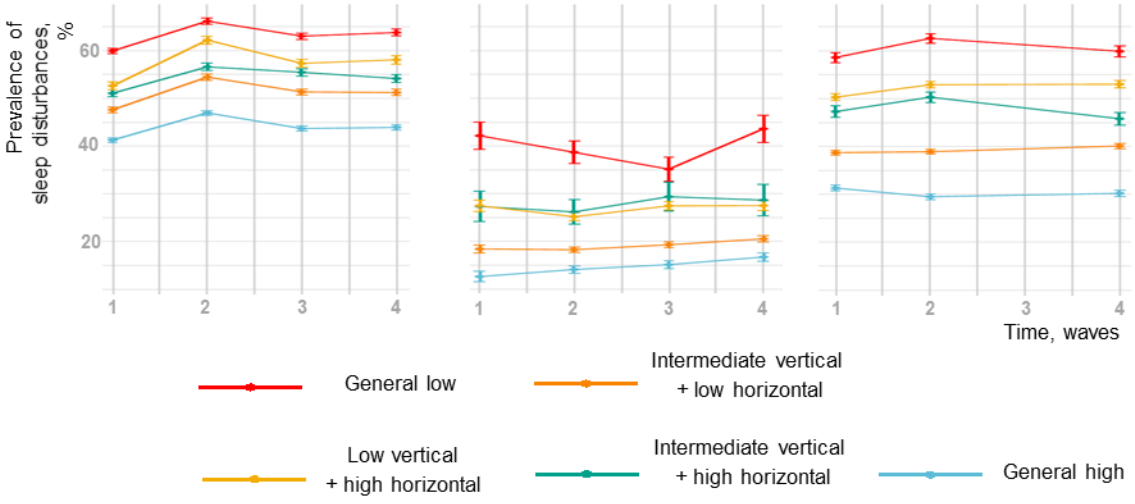

© 2023 Xu T et al. *JAMA Network Open.*

eFigure 3. Results From Analyses of Changes, Before and After the Adjustment of Night Shifts

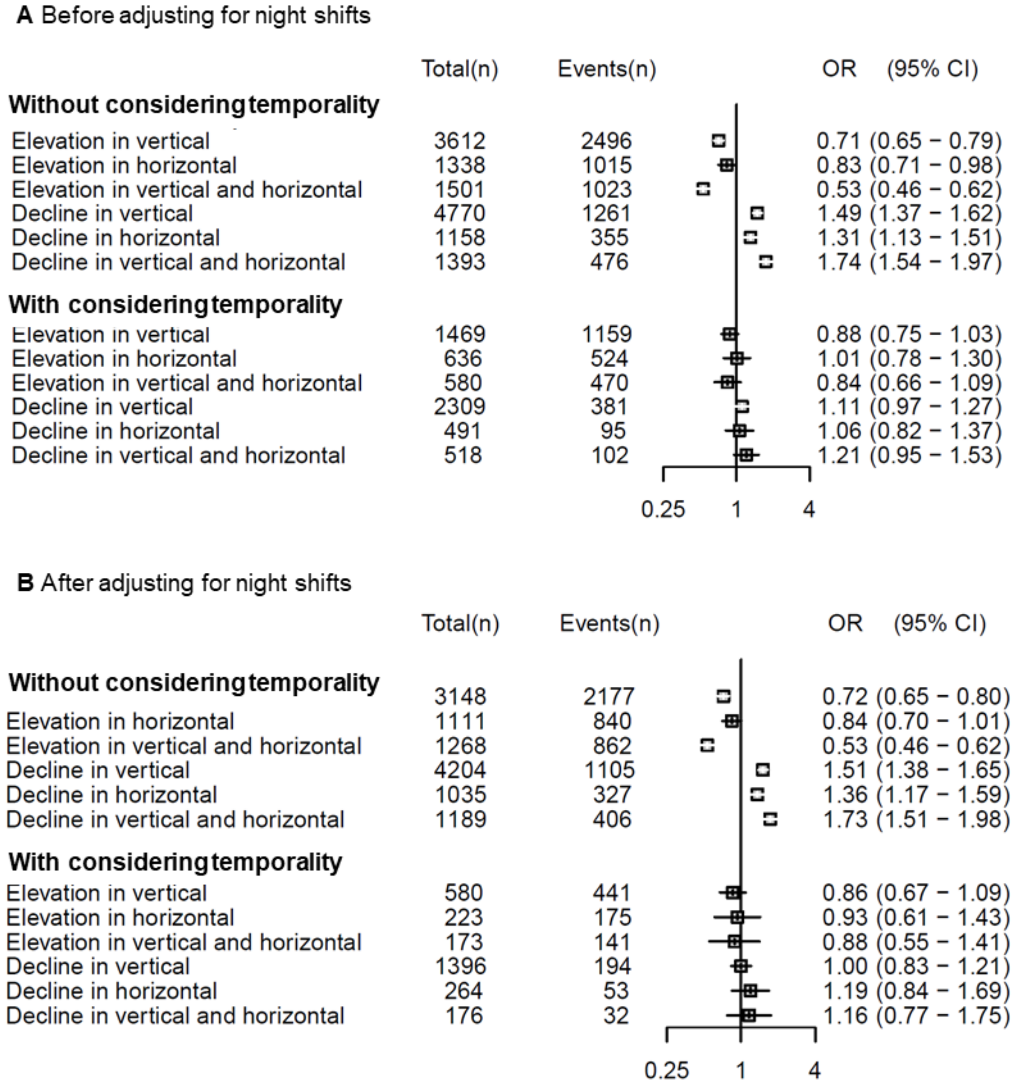

© 2023 Xu T et al. *JAMA Network Open.*

eFigure 4. Additional Adjustment for Night Shifts for Analyses on Concurrent and Longitudinal Associations

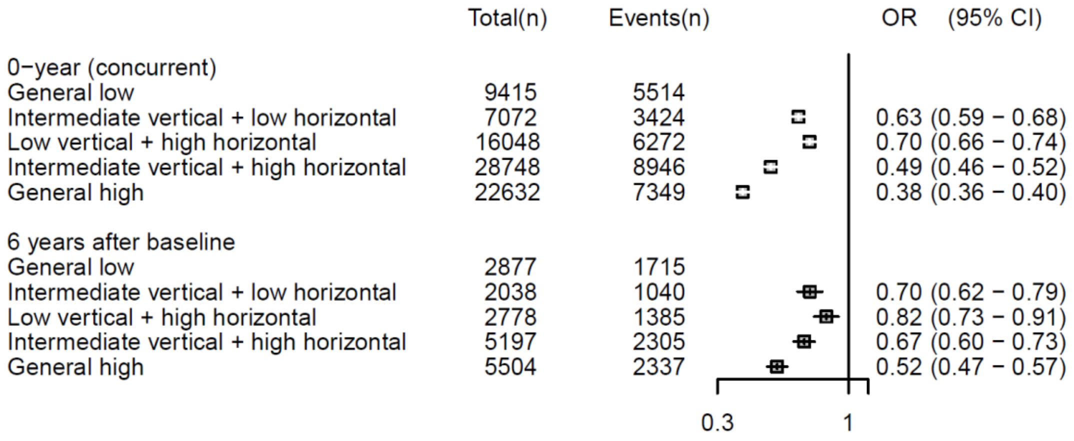

© 2023 Xu T et al. *JAMA Network Open.*

eFigure 5. Results From Alternative Definition of Sleep Disturbances

A Concurrent association

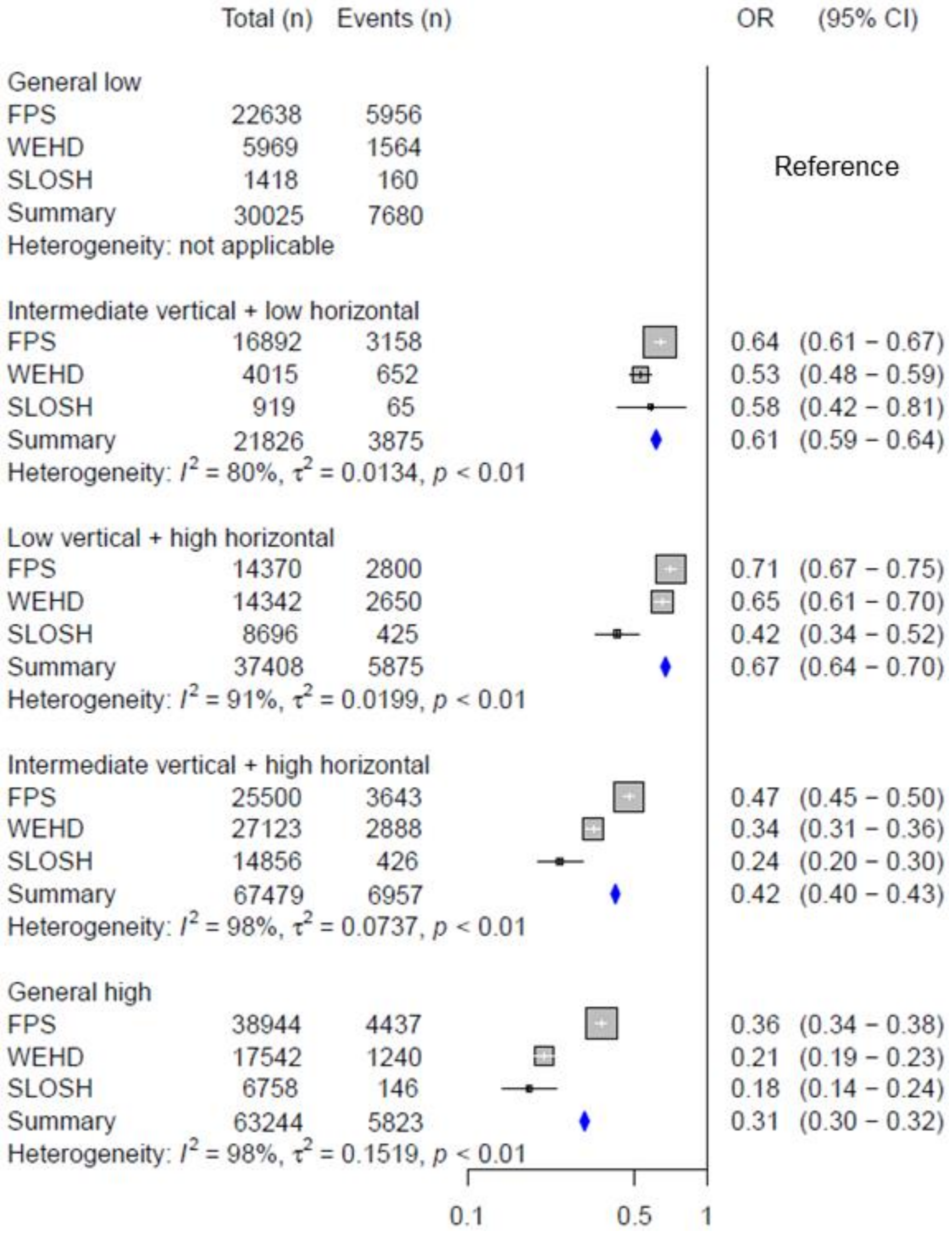

© 2023 Xu T et al. *JAMA Network Open.*

B Longitudinal association

	Total(n)	Events(n)	OR (95% CI)
0 year (concurrent)			
General low	10363	2731	Reference
Intermediate vertical + low horizontal	7720	1407	0.61 (0.56 − 0.66)
Low vertical + high horizontal	10430	1735	0.65 (0.60 − 0.71)
Intermediate vertical + high horizontal	19011	2126	0.41 (0.38 − 0.44)
General high	21397	2124	0.31 (0.29 − 0.34)
2 years after baseline			
General low	7733	1958	Reference
Intermediate vertical + low horizontal	5735	1073	0.67 (0.61 − 0.73)
Low vertical + high horizontal	7573	1233	0.69 (0.63 − 0.75)
Intermediate vertical + high horizontal	13866	1817	0.53 (0.48 − 0.57)
General high	15552	1913	0.43 (0.39 − 0.46)
6 years after baseline			
General low	2838	694	Reference
Intermediate vertical + low horizontal	1968	349	0.66 (0.57 − 0.76)
Low vertical + high horizontal	2799	467	0.71 (0.62 − 0.81)
Intermediate vertical + high horizontal	5279	673	0.53 (0.47 − 0.60)
General high	5578	713	0.47 (0.42 − 0.53)

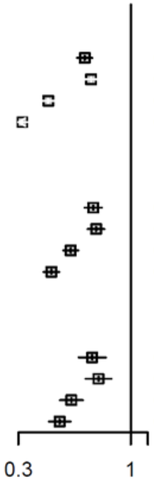

C Analysis for changes of resources

	Total(n)	Events(n)	OR (95% CI)
Without considering temporality			
Elevation in vertical	904	443	0.67 (0.56 − 0.79)
Elevation in horizontal	370	193	0.66 (0.51 − 0.85)
Elevation in vertical and horizontal	443	211	0.51 (0.41 − 0.65)
Decline in vertical	6325	582	1.55 (1.38 − 1.73)
Decline in horizontal	1711	197	1.44 (1.20 − 1.72)
Decline in vertical and horizontal	1865	276	2.50 (2.15 − 2.91)
With considering temporality	113	77	1.21 (0.74 − 2.00)
Elevation in horizontal	55	40	1.40 (0.64 − 3.06)
Elevation in vertical and horizontal	50	30	0.80 (0.38 − 1.68)
Decline in vertical	2309	110	1.07 (0.84 − 1.36)
Decline in horizontal	466	42	1.51 (1.05 − 2.18)
Decline in vertical and horizontal	388	37	1.66 (1.13 − 2.45)

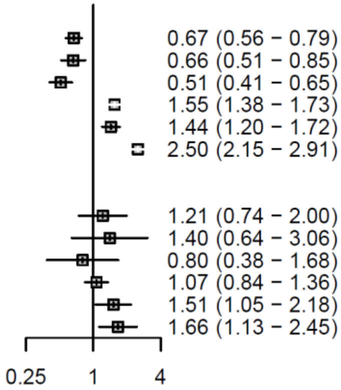

© 2023 Xu T et al. *JAMA Network Open.*
