# Supplementary material for: Workplace Psychosocial Resources and Risk of Sleep Disturbances Among Employees
Source: JAMA Netw Open. 2023 May 9;6(5):e2312514. doi: 10.1001/jamanetworkopen.2023.12514 (PMC10170336; doi:10.1001/jamanetworkopen.2023.12514)
Supplement: Supplement 2. — Data Sharing Statement [file jamanetwopen-e2312514-s002.pdf]

Data Sharing Statement

Xu. Workplace Psychosocial Resources and Risk of Sleep Disturbances Among Employees. *JAMA Netw Open*. Published May 09, 2023. doi:10.1001/jamanetworkopen.2023.12514

Data
Data available: No

Additional Information
Explanation for why data not available: This huge amount of cohort data contains rich information related to individual participants. They are not allowed to be shared publicly according to the GDPR, even unidentified. The Swedish Longitudinal Occupational Survey of Health is only accessible via Stress Research Institute in Sweden. The Work Environment and Health in Denmark study is only accessible via Statistics Denmark's server. The Finnish Public Sector Study is only accessible from Department of Public Health, University of Turku.
